# Silver nanoparticle-conjugated antibiotics inhibit *in vitro* growth of *Brucella melitensis*

**DOI:** 10.14202/vetworld.2022.1749-1752

**Published:** 2022-07-22

**Authors:** Mohamed J. Saadh

**Affiliations:** Department of Pharmacy, Faculty of Pharmacy, Middle East University, Amman, Jordan

**Keywords:** antimicrobial activity, *Brucella melitensis* Rev 1, minimum inhibitory concentrations, silver nanoparticles

## Abstract

**Background and Aim:**

Brucellosis is a contagious livestock disease with a significant economic impact. This study aimed to compare the efficacy of antibiotics used alone or in combination with silver nanoparticles (AgNPs) against *Brucella melitensis* Rev 1 *in* *vitro*.

**Materials and Methods:**

AgNps conjugated with ciprofloxacin was synthesized and thoroughly characterized by ultraviolet visible spectrophotometry (UV-vis). The antimicrobial effect of ciprofloxacin alone and ciprofloxacin conjugated with AgNPs against B. melitensis Rev 1 was determined by minimum inhibitory concentration (MIC) and the erythrocyte hemolytic assay determined the capability of conjugation to cause hemolysis in human erythrocyte.

**Results:**

The UV-vis spectra of both silver-drug nanoconjugates showed a characteristic surface plasmon resonance band at 420 nm. The MIC assays showed that AgNPs conjugation to antibiotics enhanced the antibacterial potential of the selected antibiotics against *B. melitensi*s Rev 1 relative to non-conjugated antibiotics. The results show that low concentrations of AgNPs can kill *B*. *melitensis* Rev 1. The MICs of ciprofloxacin and ciprofloxacin–AgNPs were 0.75 and 0.05 μM, respectively.

**Conclusion:**

The conjugation of ciprofloxacin with AgNPs enhanced the antibacterial effects against *B*. *melitensis* Rev 1. In addition, this conjugation appears to inhibit the capability of this bacterium to adapt to the presence of antibiotics, thereby inhibiting bacterial resistance. Further studies are required to examine its potential as an *in vivo* treatment.

## Introduction

Antimicrobial resistance has recently emerged due to the treatment of human infections [1]. Bacterial pathogens causing infectious diseases are responsible for 15 million of the 57 million annual deaths worldwide [2]. A global microbial crisis has emerged because of the increase in multidrug-resistant microbes, whereas the decrease in the efficacy of available antibiotics results from overuse [3].

Recently, nanomedicine with nanoconjugated antibiotics has shown strong antimicrobial activity against microorganisms that cause severe infections [4]. The physicochemical properties of metallic nanoparticles (NPs) have yielded extensive studies of their characteristics, including their optical, electronic, and magnetic properties in addition to their catalytic and antimicrobial activities [5]. Silver NPs (AgNPs) have been shown to effectively inhibit bacterial growth in addition to killing bacteria [6]. The larger surface areas of the AgNPs have led to improvements in antimicrobial activities relative to Ag metal alone [7].

The discovery of antibiotics has led to studies aimed at increasing the efficacy of approved antibiotics by clinically modifying these drugs or repurposing them to rapidly yield effective antibiotic formulations [6, 7]. The World Health Organization generally recommends the use of antibiotics that are sensitive to Brucella. The exponential increase in antimicrobial resistance is a very difficult and daily challenge facing by physicians [8].

This study aimed to evaluate the antibacterial activity of the synthesized AgNPs conjugated with ciprofloxacin on *Brucella melitensis* Rev 1. In addition, the AgNPs-conjugated ciprofloxacin appears to inhibit the capability of *B. melitensis* to adapt to the presence of antibiotics, thereby inhibiting bacterial resistance.

## Materials and Methods

### Ethical approval

The ethical committee approval was obtained from the Ethical Committee of Middle East University, Amman, Jordan (approval no. 2021.07).

### Study period and location

The study was conducted from January 2021 to April 2021 at Middle East University, Amman, Jordan

### Materials

*B. melitensis* Rev 1 working seed was obtained from Jordan Bio-industries Center (JOVAC, Amman, Jordan). All antibiotics, silver nitrate (AgNO_3_), and sodium borohydride (NaBH_4_) used in the current study were obtained from Sigma-Aldrich (St. Louis, MO, USA). *B. melitensis* Rev 1 experiments were conducted in accordance with biosafety level 3 guidelines [9].

### Synthesis of AgNPs coated with drugs

In brief, 5 mL of 0.1 mM of each antibiotic solution was mixed for 10 min with 5 mL of 0.1 mM of AgNO_3_ solution. A 20 μL aliquot of freshly prepared 5 mM NaBH_4_ solution was prepared and added to the reaction mixture. After the addition of a reducing agent, the color of the solution changed from transparent to yellow-brown, which indicated that the reduction of silver ions had occurred and the antibiotic–AgNPs conjugate had been formed. The AgNPs-conjugates were centrifuged at 12,000× *g* for 1 h. After lyophilization, the supernatant was collected and weighed to determine the concentration of the unconju­gated drug. The concentration of antibiotics in 100 mg of NPs was expressed as a percentage [10].

**Figure-1:**
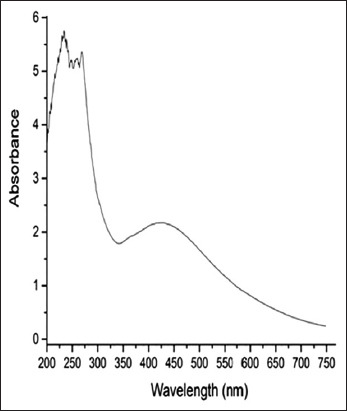
Absorption had shown of silver nanoparticles surface plasmon resonance at 420 nm.

The development of a brown color confirmed the formation of AgNPs surface plasmon resonance [11]. As shown in Figure-1, AgNPs formation was verified by the appearance of absorption peaks at 400–450 nm [10]. The percentage of drug loading on NPs was found to be 65% for ciprofloxacin–AgNps.

### Minimum inhibitory concentrations (MICs)

MICs were determined as previously described by Saadh [10]. The microdilution method was conducted in 96-well plates (Trasadingen, Switzerland) according to the Clinical and Laboratory Standards Institute recommendations.

*B. melitensis* Rev 1 was grown on Brucella Broth (Acumedia, Michigan, USA) for 18 h and then diluted to 10^6^ colony-forming units/mL in Brucella Broth. The concentrations ranged from 0.01 to 100 μg/mL for each antibiotic alone or AgNPs-conjugated antibiotics in 96-well microtiter plates with 50 mL of each concentration of antibiotic and 50 mL of diluted *B. melitensis* Rev 1. The suspension was then added to each well and the microplate was placed in the incubator at 37°C for 5 days. At the desired bacterial level of growth, the absorbance at 570 nm was measured (Thermo-lab Systems Reader, Finland).

The MIC was measured as the minimum concentration that completely inactivated bacterial growth. The positive control consisted of 50 mL of bacterial suspension + 50 mL of Brucella Broth and the negative control consisted of 100 mL of Brucella Broth in each well. The bacterial growth was assessed by direct observation of the absence of turbidity. Each test was replicated three times [10].

### Erythrocyte hemolytic assay

To determine whether AgNPs-conjugated antibiotics caused hemolysis in human red blood cells (RBCs), the hemolytic test was performed as described by Maturana *et al*. [12].

## Results and Discussion

The MIC value of ciprofloxacin was 0.75 ± 0.0047 μg/mL, as shown in Table-1. However, when this antibiotic was conjugated with AgNPs, the MIC value was 0.05 ± 0.0012 μg/mL when tested against *B. melitensis* Rev 1 (Table-1). The antibacterial results show that the ciprofloxacin–AgNPs inhibited the growth of *B. melitensis* Rev 1 *in vitro*.

**Table 1:** MIC value for ciprofloxacin and ciprofloxacin conjugated to AgNPs against *B. melitensis* Rev 1.

Antibiotics	MIC (μg/mL) ± SD	Antibiotics- AgNps	MIC (μg/mL) ± SD
Ciprofloxacin	0.75±0.0081	Ciprofloxacin–AgNps	0.05±0.0012

The MIC values were performed in triplicate. MIC=Minimum inhibitory concentration, AgNps=Silver nanoparticles, *B. melitensis=Brucella melitensis*, SD=Standard deviation

Moreover, in general, the conjugation of ciprofloxacin with AgNPs increased antibacterial efficiency compared with that of free ciprofloxacin against *B. melitensis* Rev 1. *In vitro*, hemolysis was not caused by antibiotic–AgNPs in human RBCs (Table-2).

**Table 2:** The hemolysis activity of Ciprofloxacin–AgNps in human erythrocytes *in vitro*.

Concentration (μg/mL)	Hemolysis %
50	0
80	0
100	1

AgNps=Silver nanoparticles

Antibiotic resistance shown by pathogenic bacteria is inevitable, and nanotechnology has shown great promise in developing new antibiotics to combat these bacteria. Drug delivery using nanotechnology systems has the potential to enhance drug pharmacokinetics and pharmacodynamics [13, 14]. The small size of the NPs offers a large surface area with high accessibility and maximum drug loading geared at specific targets. Recently, various drug-conjugated NPs have been used to treat antibiotic-resistant bacterial infections [14].

In this study, the prospective antibacterial activity of the synthesized AgNPs was detected and demonstrated high antibacterial activity against *B. melitensis* Rev 1. The very low MIC values of ciprofloxacin when conjugated to AgNPs indicated very good activity when compared with ciprofloxacin alone.

AgNPs can kill bacteria through different mechanisms when used as antibacterial agents. One of the mechanisms of bacterial inactivation is the interaction of Ag with proteins and amino acids, both of which contain sulfur inside and outside the bacterial cell membrane. In addition, the inhibition of enzyme activities by the Ag ions released from the AgNPs following interaction with sulfur in proteins and phosphorous in DNA [6, 15, 16]. In addition, other factors can affect antibacterial activity. It has been observed that in studies of size-dependent AgNPs, AgNPs of <20 nm have greater sulfur-containing membrane protein adhesion, which leads to maximum membrane permeability and ultimately kills bacteria [6, 15]. In contrast, AgNPs of <10 nm form pores and disrupt the cell wall, thus causing cell death without interacting with nucleic acids and proteins both inside and outside the bacterial cell. The interaction of AgNPs with some cells can trigger apoptosis [17].

The positive charge of AgNPs allows interaction with the lipopolysaccharide of Gram-negative bacteria, which AgNPs have a higher affinity for than the cell wall of Gram-positive bacteria, which has almost no interaction sites. The drug then enters the host cell [18]. In addition, AgNPs will produce reactive oxygen species (ROS), leading to microorganism death [16, 17].

The activity of AgNPs was reported against *E. coli*, *E. faecalis*, *K pneumonia*, *P. aeruginosa*, and *E. coli* [19, 20]. Levofloxacin conjugated with AgNPs exhibited antibacterial activity against methicillin-resistant *Staphylococcus aureus* [10]. *Rivina humilis*–AgNPs exhibited potential antibrucellosis activity, effectively inhibiting *Brucella abortus*, *B. melitensis*, and *Brucella suis* [21]. AgNPs were presented as a new NP drug against intramacrophage *B. melitensis* 16M [22].

Several studies have shown that AgNPs can induce cytotoxicity, genotoxicity, and inflammatory response in cells, which result in the generation of intracellular ROS, DNA damage, and the activation of signaling cascades, finally leading to cell death [23, 24]. In addition, prolonged exposure to Ag in the form of an insoluble compound leads to the deposition of silver in cell membranes and neuronal structures under the skin, which may cause skin diseases such as argyria [25].

## Conclusion

Antibiotics conjugated with AgNPs show enhanced antibacterial efficacy. The results of antibacterial assays showed that ciprofloxacin–AgNPs inhibited the growth of *B. melitensis* Rev 1 *in* *vitro*. AgNPs possess multiple mechanisms for killing bacteria. Consequently, AgNPs appear to inhibit the ability of bacteria to adapt to antibiotics, thereby inhibiting bacterial resistance. The action of these nanoparticles is not precisely understood, and it is the subject of future studies, along with testing their potential *in vivo*.

## Author’s Contributions

MJS: Designed the study, performed all the experimental procedures, preparation of AgNPs, conducted data analysis and interpretation, and drafted the manuscript. The author has read and approved the final manuscript.
